# Truncation and activation of GSK-3β by calpain I: a molecular mechanism links to tau hyperphosphorylation in Alzheimer's disease

**DOI:** 10.1038/srep08187

**Published:** 2015-02-02

**Authors:** Nana Jin, Xiaomin Yin, Dian Yu, Maohong Cao, Cheng-Xin Gong, Khalid Iqbal, Fei Ding, Xiaosong Gu, Fei Liu

**Affiliations:** 1 Jiangsu Key Laboratory of Neuroregeneration, Co-innovation Center of Neuroregeneration, Nantong University, Nantong, Jiangsu 226001, P. R. China; 2 Department of Neurochemistry, New York State Institute for Basic Research in Developmental Disabilities, Staten Island, New York 10314, USA; 3 Institute of Neurology, Department of Neurology, Hospital Affiliated to Nantong University, Nantong, Jiangsu 226001, P. R. China

## Abstract

Abnormal hyperphosphorylation of tau is pivotally involved in the pathogenesis of Alzheimer's disease (AD) and related tauopathies. Glycogen synthase kinase 3β (GSK-3β) is a primary tau kinase that is most implicated in tau pathology in AD. However, the exact molecular nature of GSK-3β involved in AD is unclear. In the present study, we found that GSK-3β was truncated at C-terminus and correlated with over-activation of calpain I in AD brain. Truncation of GSK-3β was positively correlated with tau hyperphosphorylation, tangles score and Braak stage in human brain. Calpain I proteolyzed GSK-3β *in vitro* at C-terminus, leading to an increase of its kinase activity, but keeping its characteristic to preferentially phosphorylate the protein kinase A-primed tau. Excitotoxicity induced by kainic acid (KA) caused GSK-3β truncation at C-terminus and hyperphosphorylation of tau in mouse brain. Inhibition of calpain prevented the KA-induced changes. These findings suggest that truncation of GSK-3β by Ca^2+^/calpain I markedly increases its activity and involvement of this mechanism probably is responsible for up-regulation of GSK-3β and consequent abnormal hyperphosphorylation of tau and neurofibrillary degeneration in AD.

Microtubule-associated protein (MAP) tau is abnormally hyperphosphorylated and aggregated into paired helical filaments (PHFs) or straight filaments (SFs) forming neurofibrillary tangles (NFTs) in the brains of patients with Alzheimer's disease (AD) and related tauopathies^1^,^2^. Tau is the major neuronal MAP, the biological activity of which is regulated by its degree of phosphorylation. However, the hyperphosphorylation not only destroys its biological activity but also converts it into a cytotoxic protein that sequesters the MAPs^3^,^4^,^5^.

Glycogen synthase kinase 3β (GSK-3β) is a proline-directed serine/threonine protein kinase and phosphorylates tau protein at most of the Ser/Thr-Pro sites seen in PHF-tau *in vitro* and in cultured cells^6^,^7^,^8^. The kinase activity of GSK-3β is related tightly to maintenance of cell architecture, gene expression and apoptosis, and is controlled by its phosphorylation at Ser 9 residue^9^. GSK-3β is highly expressed in the normal brain and associates with microtubules^10^. Overexpression of GSK-3β in transgenic mice results in hyperphosphorylation of tau^11^,^12^,^13^, and treatment of mice with GSK-3β specific inhibitor lithium dramatically attenuates tau phosphorylation and rescues tau-induced neurodegeneration^14^,^15^,^16^,^17^,^18^. Thus, GSK-3β is believed to play a critical role in abnormal hyperphosphorylation of tau and neurodegeneration in AD. However, to date, direct evidence and mechanism of the up-regulation of GSK-3β in AD brain have not been reported.

Calpain is a family of calcium-activated intracellular cysteine proteases that catalyzes limited proteolytic cleavage of a variety of cellular proteins in eukaryotes^19^. Calpain I, the major calpain isoform in the neuron, is present principally as an inactive precursor and is activated by autoproteolytic cleavage of the N-terminus when stimulated by low micromolar (μM) concentrations of calcium (hence, it is also called μ-calpain). Altered brain calcium homeostasis as well as truncation and activation of calpain I has been reported in AD brain^20^,^21^,^22^,^23^.

To understand the molecular nature of the involvement of GSK-3β and calpain I in the abnormal hyperphosphorylation of tau, we investigated the relationship between GSK-3β and calpain I *in vitro* and in autopsied AD and control brains. We found that GSK-3β was truncated in AD brain, which was correlated with the activation of calpain I. Calpain I proteolytically cleaved GSK-3β and enhanced its kinase activity toward tau. Excitotoxicity induced by kainic acid (KA) caused activation of calpain and GSK-3β truncation and tau phosphorylation at Ser 396 in the mouse brain. The truncation of GSK-3β was highly correlated with tau phosphorylation in human brains. These data suggest that the truncation of GSK-3β by calpain I may contribute to the hyperphosphorylation of tau and neurofibrillary degeneration in AD.

## Results

### GSK-3β is truncated in AD brain and the level of truncated kinase correlates to the level of activated calpain I

**Figure 1:**
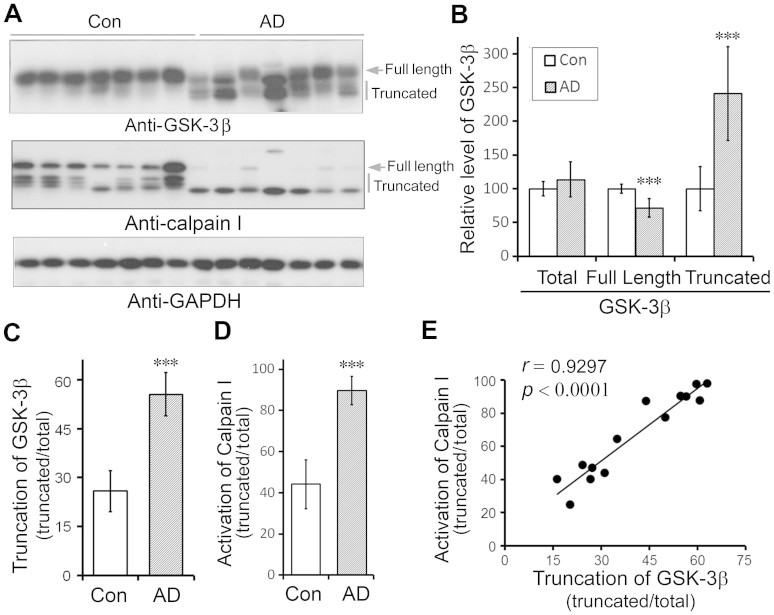
Activation of calpain I and truncation of GSK-3β are elevated in AD brain and truncation of GSK-3β is correlated with the activation of calpain I in human brain. (A) Western blots of frontal cortical homogenates from AD and control cases show an increase in truncation of GSK-3β and calpain I in AD. Arrow indicates the full-length GSK-3β or calpain I and vertical bars indicate the truncated forms of these proteins. (B) The levels of full length and truncated GSK-3β were decreased and increased, respectively, in AD brains. Blots from panel A were quantitated by densitometry and the relative levels of total, full-length and truncated GSK-3β are presented as mean ± S.D. (n = 3). ***, *p* < 0.001. (C, D) Truncation of GSK-3β and calpain I normalized to corresponding total protein levels show increase in AD brain. Data are presented as mean ± S.D. (n = 7). ***, *p* < 0.001. (E) Activation of calpain I (truncated/total) directly correlates with the truncation of GSK-3β (truncated/total) in human brain. The Spearman correlation coefficient *r* and *p* value are shown.

To understand the role of GSK-3β in tau pathogenesis in AD, we determined the expression of GSK-3β in frontal cortices from 7 AD and 7 age- and postmortem interval–matched control brains that were obtained ≤3.5 h after death (Table S1) by Western blots developed with R127, an antibody against residues 364–377 of GSK-3β (referred to the longest isoform). We observed two major bands (47-kDa and 41-kDa) of GSK-3β in AD cases, but mainly the 47-kDa band in control cases (Fig. 1A). The total protein level of GSK-3β in AD cases was similar to that in control cases, whereas the full-length GSK-3β band was dramatically reduced, and the truncated GSK-3β was markedly increased in AD brains (Fig. 1A, B). Thus, the truncation of GSK-3β was increased markedly in AD brain (Fig. 1A, C).

To learn whether the truncation of GSK-3β is resulted from postmortem delay, we first analyzed the difference in postmortem interval (PMI) between AD and Control cases and the relationship of PMI with its truncation. We found that the PMI in AD group was shorter than in control group, but did not reach statistical difference (Fig. S1A). Also, there was no significant correlation between GSK-3β truncation and PMI (Fig. S1B). To further rule out the PMI as a cause of GSK-3β truncation, we kept the mouse bodies up to 3 hr at room temperature or at 4°C after death to mimic postmortem delay, and then analyzed GSK-3β by Western blots. We did not observe any truncation in GSK-3β during 3 hr PMI (Fig. S1C). These results suggest that PMI up to 3 hr does not cause truncation of GSK-3β. Thus, truncation of GSK-3β in AD brains we observed probably represented pathology and not a postmortem artifact.

Calpain I is autoproteolyzed and activated in AD brain. We analyzed calpain I in brain homogenates by Western blots and found that consistent with previous reports, calpain I, an 80-kDa full length protein, was truncated to the 78/76-kDa active forms in AD brain (Fig. 1A, D)^20^,^23^. To learn the relationship between truncation of GSK-3β and the over-activation of calpain I, we analyzed the correlation between them in human brain homogenates. We observed that truncation of GSK-3β was correlated with activation of calpain I strongly and positively (*r* = 0.9297, *p* < 0.0001) (Fig. 1E), suggesting that overactivation of calpain I might have led to GSK-3β truncation in AD brain.

### Truncation of GSK-3β is positively correlated with hyperphosphorylation and pathology of tau

**Figure 2:**
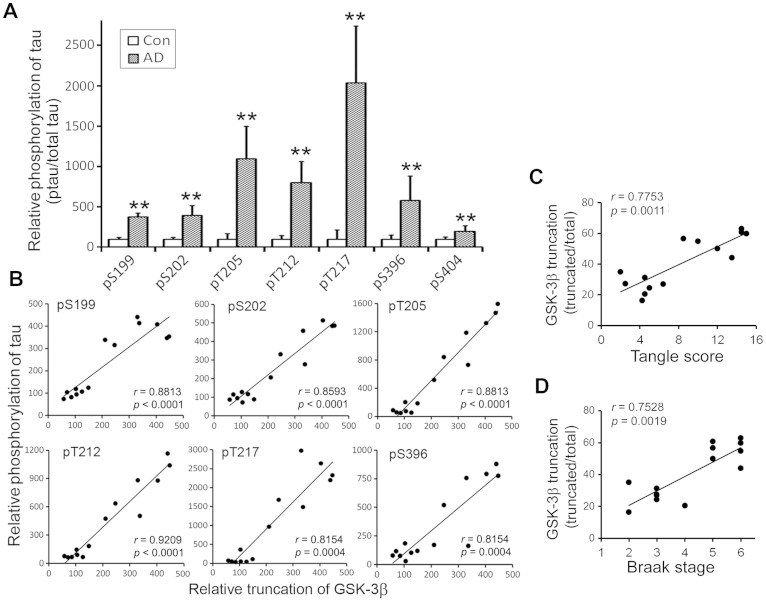
Truncation of GSK-3β is positively correlated with site-specific phosphorylation of tau, Tangle Score and Braak State in human brain. (A) Quantitation of immune-dot-blots (not shown) by densitometry shows hyperphosphorylation of tau at each site in AD brain. Tau phosphorylation at individual phosphorylation sites in the frontal cortical crude extracts from 7 AD and 7 control cases were determined by quantitative immuno-dot-blots and relative signal obtained by densitrometry is shown as mean ± SD (n = 7). **, *p* < 0.01. (B) Spearman correlation analysis shows a direct correlation of truncation of GSK-3β with tau phosphorylation at various sites. The levels of tau phosphorylation at individual phosphorylation site in AD and control brains same as in panel A (Y-axis) were plotted against the truncation of GSK-3β (ratio of truncated over full-length GSK-3β) (X-axis) from Fig. 1C. (C, D) Spearman correlation analysis shows correlation of truncation of GSK-3β with Tangle Score and Braak Stage. Truncation of GSK-3β from Fig. 1C (Y-axis) were plotted against the Tangle score (C) or Braak Stage (D) (X-axis) from Table S1.

To determine the relationship between GSK-3β and tau phosphorylation in human brain, we measured tau phosphorylation levels at individual sites in the frontal cortex from 7 AD and 7 control brain cases and analyzed the correlation of GSK-3β truncation with phosphorylation of tau. We found that, as expected, the abnormal phosphorylation of tau at many sites was significantly increased in AD brain (Fig. 2A). The truncation of GSK-3β was positively correlated with tau phosphorylation at Ser 199, Thr 202, Thr 205, Thr 212, Thr 217, and Ser 396 (Fig. 2B). These results support the contribution of the truncation/activation of GSK-3β to hyperphosphorylation of tau in AD brain.

NFTs are made up of hyperphosphorylated tau. Both Braak stage and CERAD Alzheimer's Disease Criteria tangle score are the methods used to classify the degree of tau pathology in AD^24^,^25^. Normal aged human brain has limited numbers of NFTs which is classified as Braak stage II–IV^26^. To determine the relationship of GSK-3β with tangle score and Braak stage, we analyzed their correlation. We found that truncation of GSK-3β was positively correlated with the Braak stage and tangle sore (Fig. 2C, D). These results support the contribution of the truncation/activation of GSK-3β to tau pathology in AD brain.

Truncation of GSK-3β is caused by activation of calpain I. We determined the relationship of calpain I activation and tau phosphorylation. We also observed a significant and positive correlation between the activation of calpain I and tau phosphorylation at many sites (Fig. S2). These results suggest that truncation/activation of calpain I relates to tau hyperphosphorylation in AD brain.

### GSK-3β is truncated by calpain I

**Figure 3:**
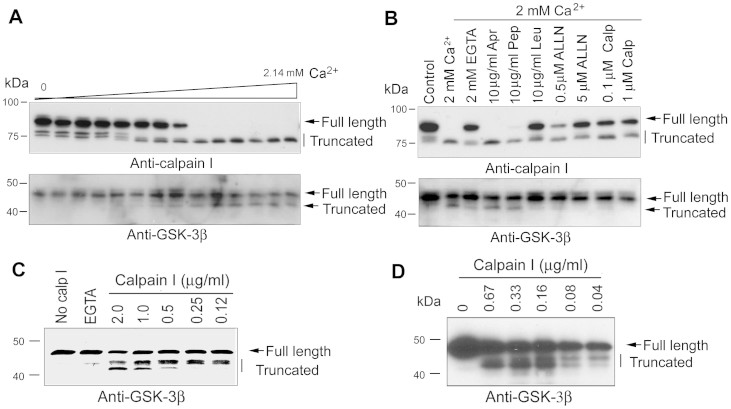
GSK-3β is truncated selectively by calcium-mediated truncation/activation of calpain I. (A) Western blots show that truncation of GSK-3β coincides with truncation/activation of calpain I in a calcium-dependent manner in human brain extracts. Normal human brain extract was incubated at 30°C for 10 min in the presence of 2.0 mM EDTA and various concentrations (0.00–2.14 mM) of CaCl_2_. Then the incubated extracts were analyzed by Western blots developed with specific antibodies to calpain I or GSK-3β. (B) Calcium-activated truncations of calpain I and GSK-3β are selectively inhibited by calpain inhibitors. Normal human brain extract was incubated at 30°C for 10 min in the presence of 2.0 mM each of EDTA and CaCl_2_ plus various selective protease inhibitors, as indicated above the blots, followed by Western blots probed with anti-calpain I or anti-GSK-3β to detect the proteolysis. Apr, aprotinin (a serine protease inhibitor); Pep, pepstatin (an aspartic protease inhibitor); Leu, leupeptin (cysteine and serine protease inhibitor that also inhibits calpain); ALLN, N-acetyl-Leu-Leu-Nle-CHO (a cysteine protease inhibitor that also inhibits calpain); Calp, calpastatin peptide (a specific calpain inhibitor). (C, D) Western blots show calcium concentration-dependent truncation of recombinant GSK-3β from bacteria (C) or from mammalian cells (D) by calpain I. Recombinant GSK-3β was incubated with various concentration of calpain I in the presence of CaCl_2_ for 10 min at 30°C and the reaction products were subjected to Western blots.

To learn whether activation of calpain I is responsible for the truncation of GSK-3β, we performed calcium-dependent proteolysis by incubating normal human brain extract in the presence various concentrations of calcium together with 2.0 mM EDTA at 30°C for 10 min. After terminating the reaction by boiling the samples in Laemmli buffer, calpain I and GSK-3β were analyzed by Western blots. We found that calpain I was truncated from 80-kDa into 78-kDa and further into 76-kDa active forms in a Ca^2+^ dose–dependent manner (Fig. 3A). Interestingly, GSK-3β was truncated into the 43-kDa and further to 41-kDa Ca^2+^ concentration–dependently, which coincided with the truncation/activation of calpain I (Fig. 3A), suggesting that GSK-3β is truncated by a calcium-dependent protease(s) in human brain extract.

Calpain is a protein belonging to the family of calcium-dependent, non-lysosomal cysteine proteases. To examine the calcium-dependent proteolysis of GSK-3β and calpain I in the human brain, we studied the inhibition of the Ca^2+^-stimulated proteolysis with various selective protease inhibitors. When aprotinin, a serine protease inhibitor, and pepstatin, an aspartic protease inhibitor, were added to the normal human brain extracts during incubation, neither aprotinin nor pepstatin significantly inhibited the Ca^2+^-induced truncation of calpain I or GSK-3β (Fig. 3B). These results excluded serine proteases and aspartic proteases from the involvement of the proteolysis of calpain I and GSK-3β. Leupeptin and N-acetyl-Leu-Leu-Nle-CHO (ALLN) (cysteine and serine protease inhibitors) inhibited the proteolyses of calpain I and GSK-3β (Fig. 3B). Furthermore, a specific calpain inhibitor, calpepstatin peptide, also inhibited the auto-truncation of calpain I and prevented GSK-3β from proteolysis (Fig. 3B). Thus, these results indicate that calpain is responsible for the truncation of GSK-3β in brain. Because of the presence of 2.0 mM EDTA that chelated most of Ca^2+^, only micromolar, not millimolar, levels of free Ca^2+^ were present in the reaction mixture (Fig. 3A), which suggest that GSK-3β proteolysis observed in the human brain extracts most likely results from the activation of calpain I, rather than calpain II, the latter of which requires mM concentrations of free Ca^2+^ for activation. Taken together, these results suggest that probably the elevated Ca^2+^ activates calpain I, which in turn cleaves GSK-3β into the 43-kDa and 41-kDa truncated form in the brain.

In order to confirm that calpain I proteolyzes GSK-3β, we incubated recombinant GSK-3β with calpain I in the presence of calcium. We observed that GSK-3β was cleaved by calpain I dose-dependently. Low concentration of calpain I cleaved GSK-3β into the 43-kDa fragment and higher concentration of calpain I cleaved it into the 43- and 41-kDa fragments (Fig. 3C).

Usually, proteins are post-translationally modified in eukaryotic cells. To investigate the potential effect of post-translational modifications, we studied the proteolysis of HA-GSK-3β immune-purified from HEK-293FT cells with calpain I. We incubated HA-GSK-3β with various concentrations of calpain I in the presence of CaCl_2_ for 10 min and analyzed the proteolyzed products by Western blots. We found that similar to recombinant GSK-3β from *E.* coli (Fig. 3C), calpain I also cleaved GSK-3β purified from mammalian cells effectively and dose-dependently (Fig. 3D), suggesting that phosphorylation does not block the proteolysis of GSK-3β by calpain I.

Calpains I and II are well known to proteolyze the same substrates but require μM and mM calcium, respectively. To learn whether calpain II also proteolyze GSK-3β as calpain I, we performed *in vitro* preteolysis of immune-purified GSK-3β from HEK-293FT cells with calpains I and II. We found that both calpains I and II proteolyzed GSK-3β similarly (Fig. S3).

### Truncation of GSK-3β by calpain I enhances its kinase activity

**Figure 4:**
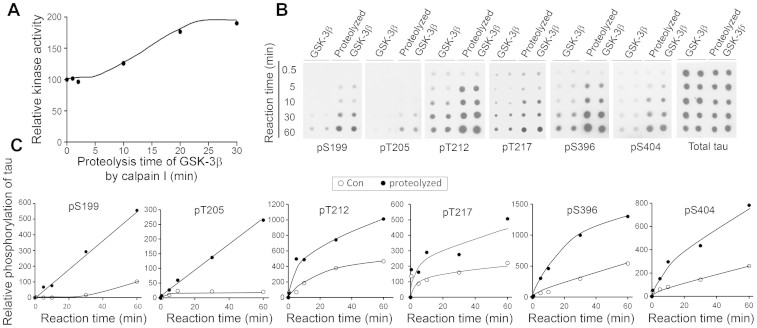
Proteolysis of GSK-3β by calpain I increases its tau kinase activity. (A) Proteolysis of GSK-3β by calpain I elevates its kinase activity toward tau in a time dependent manner. Recombinant GSK-3β was incubated with 0.1 μg/ml calpain I for various times at 30°C. The reaction products were subjected to kinase activity assay toward tau_441_ in presence of a calpain inhibitor, ALLN. (B, C) Immuno-dot-blots developed with phospho-dependent antibodies to various phosphorylation sites of tau and anti-total tau show that proteolysis of GKS-3β by calpain I increases its kinase activity. Recombinant GSK-3β from mammalian cells was incubated without or with 0.2 μg/ml calpain I in presence of 1 mM CaCl_2_ for 10 min at 30°C. The reaction products were incubated with tau_441_ for various times (0.5–60 min) in the presence of ALLN. The phosphorylation of tau at individual sites was assayed by immune-dot blots (B) and relative level of tau phosphorylation at individual sites detected in panel B was quantitated by densitometry and plotted against the reaction time (0–60 min) (C).

To learn the impact of the truncation of GSK-3β by calpain I on its kinase activity, we assayed the kinase activity of GSK-3β toward tau_441_ after incubation with calpain I for various periods of time. We found that proteolysis of GSK-3β by calpain I increased its kinase activity in a proteolysis time–dependent manner (Fig. 4A). Proteolysis of GSK-3β with 0.5 μg/ml calpain I for 20-30 min increased its kinase activity by ~2 fold (Fig. 4A).

GSK-3β phosphorylates tau at multiple sites differentially^27^. To examine if the proteolysis of GSK-3β by calpain I increases its tau kinase activity site-specifically, the immuno-purified HA-GSK-3β from HEK-293FT cells was proteolyzed with 2 μg/ml calpain I for 10 min at 30°C. Under this condition, more than half of HA-GSK-3β was proteolyzed into the 41-kDa fragment (Fig. S4A). The proteolyzed and control-treated HA-GSK-3β were used to phosphorylate tau for various periods of time. We analyzed tau phosphorylation at individual sites by immuno-dot blots. We found that the proteolyzed GSK-3β was more effective in phosphorylation of tau, but to different levels, at Ser 199, Thr 205, Thr 212, Thr 217, Ser 396 and Ser 404 than the control-treated GSK-3β (Fig. 4B, C). Proteolyzed GSK-3β phosphorylated tau at Ser 199, Thr 205, Thr 212, Thr 217, Ser 396 and Ser 404 almost 6-, 11-, 2.5-, 3-, 2.5- and 4-fold more than the control-treated kinase (Fig. 4C). The levels of phosphorylated Ser 9 GSK-3β were similar between con- and proteolysed-GSK-3β (Fig. S4B), suggesting that the truncation increases the activity of GSK-3β independently from its Ser 9 phosphorylation.

### Calpain I truncated GSK-3β keeps its kinase priming characteristic

**Figure 5:**
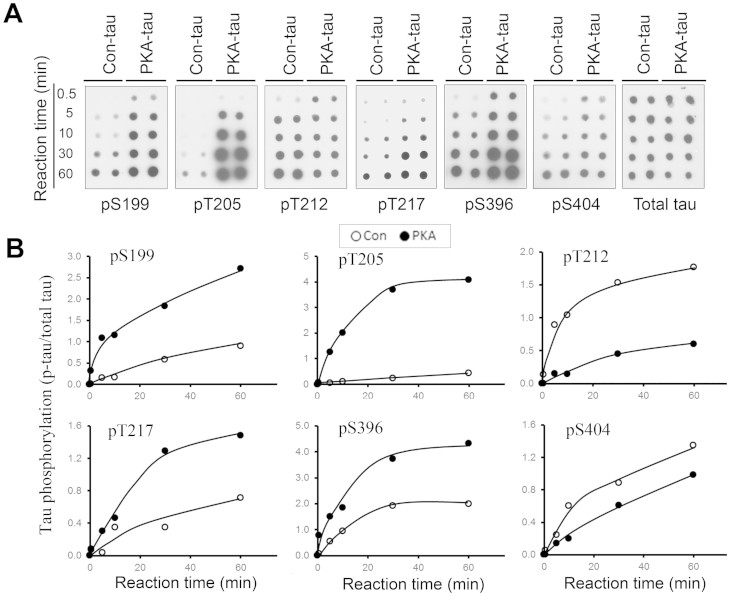
Truncation of GSK-3β affects its ability to phosphorylate PKA-primed tau in site-specific manner. Recombinant GSK-3β was first incubated without or with 0.2 μg/ml calpain I in the presence of 1 mM CaCl_2_ for 10 min at 30°C. The GSK-3β truncation by calpain I was inhibited by adding ALLN, followed by incubation with tau_441_ or PKA-prephosphorylated tau_441_ for various times. The phosphorylation of tau at individual sites was measured by immuno-dot blots developed with phospho-dependent antibodies to various specific phosphorylation sites of tau (A) and the relative levels of tau phosphorylation at individual sites detected in panel A were quantitated by densitometry and plotted against tau phosphorylation reaction times (B). Truncation of GSK-3β by calpain I enhanced its kinase activity toward tau Ser 199, Thr 205, Thr 217 and Ser 396, but like full length GSK-3β (see Fig. S5) the ability of the truncated kinase to phosphorylate PKA-phosphorylated tau at Thr 212 is reduced.

Previously we showed that PKA primes GSK-3β-catalyzed tau phosphorylation^27^. To learn whether the proteolyzed GSK-3β preserves this characteristic, we phosphorylated PKA-phosphorylated tau (PKA-tau) with truncated or control-treated GSK-3β for various times and then determined the phosphorylation level at each site by immuno-dot blots. We found that like the full-length GSK-3β (Fig. S5), the proteolyzed GSK-3β also phosphorylated PKA-tau considerably more efficiently than control-treated tau at several sites, including Ser 199, Thr 205, Thr 217, Ser 396, but not Thr 212; priming of tau by PKA inhibited its phosphorylation at Thr 212 by GSK-3β (Fig. 5).

### Calpain I cleaves GSK-3β at C-terminus

**Figure 6:**
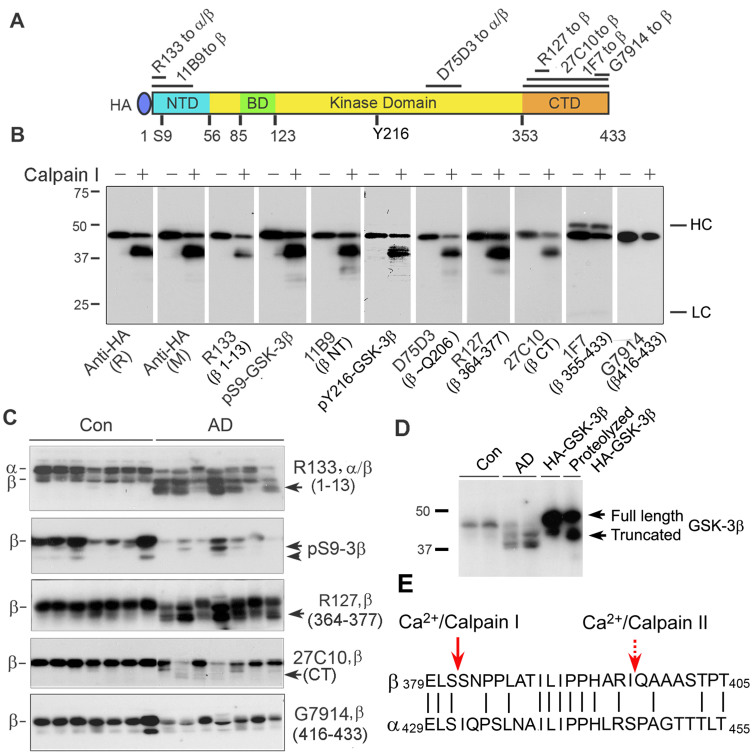
GSK-3β is truncated at the C-terminus between Ser381 and Ser382 by calpain I *in* *vitro* and in AD brain. (A) Schematic of GSK-3β domain structures and the epitopes of antibodies used to map the truncation. (B) Western blots of GSK-3β proteolysis by calpain I and of control (no calpain I) developed with various antibodies to N-terminal and C-terminal of GSK-3β. Recombinant GSK-3β was incubated without or with 0.2 μg/ml calpain I in the presence of 1 mM CaCl_2_ for 10 min at 30°C. The proteolyzed products were analyzed by Western blots with a series of antibodies against different epitopes of GSK-3β. NT, N-terminal region; CT, C-terminal region; HC, Ig G heavy chain; LC, Ig G light chain. (C) Western blots of AD and control brain homogenates developed with various N-terminal and C-terminal anti-GSK-3β. Western blots of frontal cortical homogenates from AD and control cases developed with antibodies against different epitopes of GSK-3β, as labeled in panel A. Arrow indicates truncated GSK-3β; arrow head indicates a non-AD-related truncation of GSK-3β. (D) Western blots of AD and control brain homogenates and of HA-GSK-3β proteolyzed by calpain I in vitro and its control developed with R127. Western blots of frontal cortical homogenates from AD, control cases, and immunpuified GSK-3β proteolyzed with or without calpain I as described above developed with antibody R127 to GSK-3β. (E) Amino acid sequences of regions of GSK-3β (β) and GSK-3α (α) where calpain I cleaves GSK-3β (between Ser 381 and Ser 382). GSK-3β-GST recombinant fusion protein was proteolyzed with calpain I *in vitro*, then subjected to SDS-PAGE and the truncated GSK-3β band recognized by anti-GST was cut out and subjected to N-terminal sequencing. Dotted arrow shows the truncation site between Ile 397-Gln 398 reported in the literature for calpain II^29^.

To map the cleavage region/site(s) of GSK-3β, we proteolyzed the immuno-purified HA-GSK-3β with calpain I in the presence of calcium for 10 min. The proteolyzed product was analyzed by Western blots developed with a series of antibodies against the different epitopes of GSK-3β (Fig. 6A). We observed that both uncleaved and cleaved GSK-3β immunoreacted with all anti-N-terminal GSK-3β antibodies (R133, 11B9 and anti-pSer9-GSK-3β) and anti-middle region of GSK-3β antibodies (D75D3, R127 and 27C10), but the cleaved enzyme was not immunolabeled with anti-C-terminal GSK-3β, 1F7 and G7914 (Fig. 6B). These data indicate that the cleavage site(s) are located at the C-terminus, but not at N-terminus, of GSK-3β.

GSK-3β was previously reported to be cleaved by calpain at both the N-terminus (Thr 38-Thr 39) and/or the C-terminus (Ile 397-Gln 398)^28^,^29^. However, in the present study, we did not find the N-terminal cleavage of HA-GSK-3β immunopurified using anti-HA. To rule out the possibility that the binding of anti-HA to HA-GSK-3β may interfere with the N-terminal proteolysis, we added GST to the C-terminus of GSK-3β and affinity-purified it with glutathione-sepharose. Then, we proteolyzed GSK-3β-GST with calpain I, as described above, and analyzed the proteolysis by Western blots. We found that, like HA-GSK-3β, GSK-3β-GST was truncated only at C-terminus, but not at N-terminus (Fig. S6).

To determine the truncation region/sites of GSK-3β in AD brain, we analyzed GSK-3β in human brain with different antibodies. We observed that like the GSK-3β truncated *in vitro* by calpain I, the truncated form of GSK-3β in human brain was recognized by N-terminal antibodies, R133 and anti-pS9-GSK-3β, and the middle region antibodies, R127, but not by the C-terminal antibody G7914 (Fig. 6C). These data suggest that similar to *in vitro* cleavage by calpain I, the GSK-3β in AD brains was truncated between a.a. 377 to a.a. 416. The truncated GSK-3β in vitro by calpain I was slightly bigger than GSK-3β in AD brain because immunopurified GSK-3β from HEK-293FT cells was tagged by HA at its N-terminal, (Fig. 6D). Nevertheless, calpain I proteolyzed GSK-3β to generate AD-like truncation.

To identify the cleavage site(s) of GSK-3β by calpain I, we proteolyzed GSK-3β-GST fusion protein by calpain I and separated the products by SDS-PAGE. The truncated GSK-3β band recognized by anti-GST was subjected for N-terminal sequencing. We found that the amino acid sequence started with Ser-Gln (SN), suggesting GSK-3β was cleaved by calpain I at Ser 381-Ser 382 (Fig. 6E). This cleavage site is slightly upstream of Ile397-Gln398 previously reported for calpain II^29^.

### Truncated GSK-3β has higher ability to phosphorylate tau

**Figure 7:**
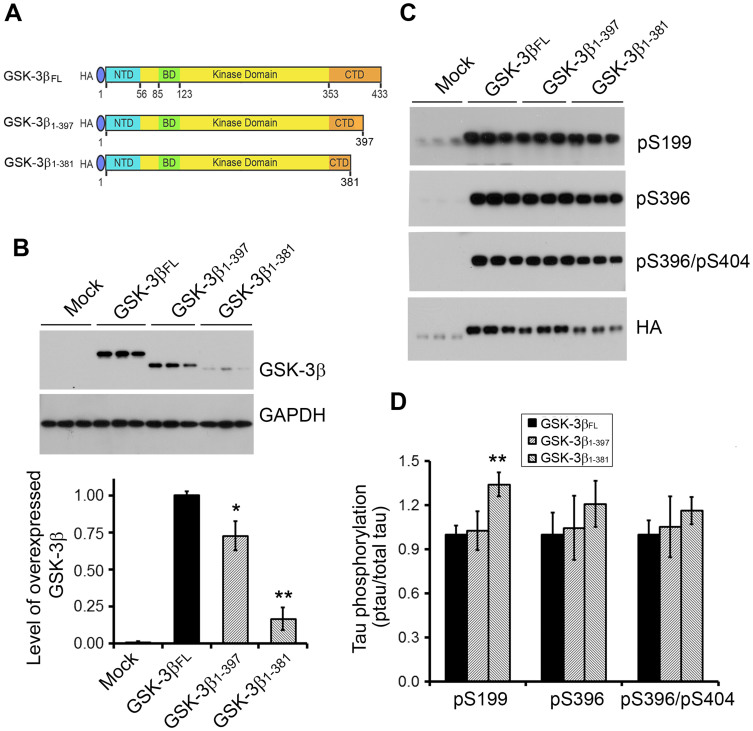
Truncation of GSK-3β at Ser381 enhances its tau kinase activity. (A) Schematics of GSK-3β full legth and C-terminally truncated at Ile 397 or Ser 381. (B) Western blots of full length and truncated GSK-3β overexpressed in HEK-293FT cells. pCI/HA-GSK-3β, pCI/HA-GSK-3β_1-397_ or pCI/HA-GSK-3β_1-381_ was co-transfected with pCI/tau_441_ into HEK-293FT cells. The levels of GSK-3β and actin were analyzed by Western blots, quantified by densitometry and normalized by GAPDH. The data are presented as mean ± S.D. (n = 3). **, *p* < 0.01. (C) Western blots of cell lysates from (B) developed with phosph-dependent tau antibodies to specific phosphorylation sites. Phosphorylation of tau at Ser199, Ser396 and Ser404 in cells from B was determined by Western blots with phosphorylation dependent- and site-specific tau antibodies and (D) the levels of phosphorylated tau determined by densitometry of the blots was normalized by total detected by anti-HA and presented as mean ± S.D. (n = 3).

GSK-3β is a major tau kinase and phosphorylates tau at many sites, but Ser 396 and Ser 199 are its preferred sites^27^,^30^. In order to compare the tau kinase activities of the calpain I- and II-truncated GSK-3β with the full length protein, we co-overexpressed HA tagged full length GSK-3β (GSK-3β_FL_) or its C-terminal truncated forms, GSK-3β_1-397_ and GSK-3β_1-381_ (Fig. 7A), with tau in HEK-293FT cells, and then measured tau phosphorylation by Western blots. We observed that the levels of GSK-3β_1-397_ and GSK-3β_1-381_ in transfected cells were 75% and 20% of full length GSK-3β (Fig. 7B, C), but phosphorylation levels of tau at Ser 199 and Ser 396 in these cells were similar to or little higher than that in GSK-3β_FL_ expressed cells (Fig. 7D). These results suggest that C-terminal truncation forms of GSK-3β, specially GSK-3β_1-381_, have higher activity than GSK-3β_FL_ in phosphorylation of tau.

### GSK-3β is truncated in the kainic acid-induced excitotoxic brain

**Figure 8:**
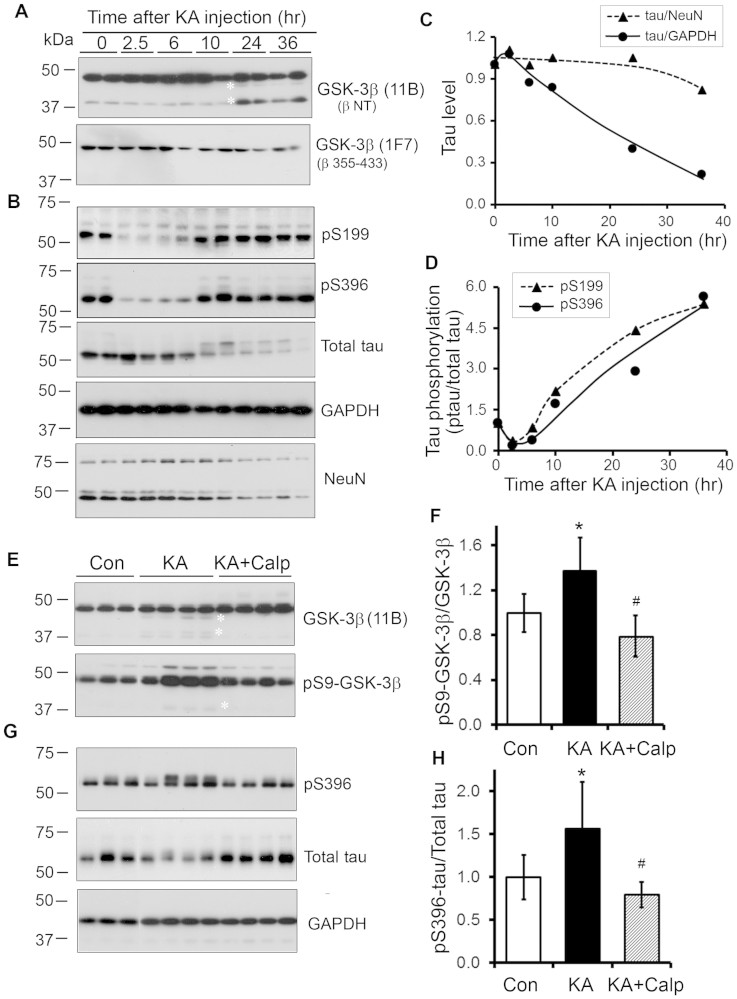
Kainic acid (KA)-induced excitotoxicity causes truncation of GSK-3β and an increase in tau phosphorylation in mouse brain. (A, B) Western blots show C-terminal truncation of GSK-3β (A) and hyperphosphorylation of tau (B) in brains of mice intraperitoneally injected with KA. Homogenates of forebrains of mice sacrificed at the indicated time points after single intraperitoneal KA injection were analyzed by Western blots developed with GSK-3β antibodies indicated at right side of each blot. (C, D) KA-induced excitotoxicity increased phosphorylation of tau at Ser 199 and Ser 396. Forebrain homogenates of KA-injected mice were analyzed by Western blots (B) developed with anti-pSer199, PHF-1 (pS396), anti-total-tau, anti-NeuN and anti-GAPDH. The levels of total tau (C) were normalized with NeuN or GAPDH, and Phosphorylation of tau at Ser 199 and Ser 396 (D) were normalized with total tau level and plotted against the time after KA-injection. The data are presented as mean of two mice. (E, F) Inhibition of calpain prevents kainic acid (KA)-induced truncation and phosphorylation of GSK-3β in mouse brains. Calpain inhibitor calpeptin was intracerebroventricularly injected 3 hr before KA intraperitoneal injection. Homogenates of forebrains of mice sacrificed after 12 hr KA injection were analyzed by Western blots developed with GSK-3β and quantified densitometrically. The level of pSer9-GSK-3β (normalized with GSK-3β) (F) is shown as mean ± S.D. (n = 4–6). (G, H) Inhibition of calpain suppressed tau phosphorylation at Ser396 caused by KA even though inhibitory phosphorylation of GSK-3β at Ser 9 was decreased. Total tau and phosphorylated tau at Ser 396 in above samples were analyzed by Western blots (G) and quantified as described above. The level of phosphorylated tau at Ser 396 normalized with total tau (H) is shown as mean ± S.D. (n = 4–6); *, KA Via Con and *p* < 0. 05; #, KA+Calp via KA and *p* < 0. 05. *, indicates truncated GSK-3β.

Excitotoxicity induces calcium influx and calpain I activation^31^,^32^. To study GSK-3β is truncated by calpain I in vivo, we injected kainic acid (KA) intraperitoneally, which is a commonly used method to induce excitotoxicity, and measured GSK-3β truncation. We found a time-dependent truncation of GSK-3β at ~40 kDa after KA injection and truncated GSK-3β could be immunostained by anti-N-terminal GSK-3β, but not by anti-C-terminal GSK-3β (Fig. 8A), suggesting that GSK-3β is truncated at C-terminal in excitotoxic brain.

To determine the effect of truncation and activation of GSK-3β by excitotoxicity on tau phosphorylation, we measured total tau and tau phosphorylation at Ser 199 and Ser 396, the two most favorite sites of GSK-3β^33^, in KA-induced excitotoxic brain. We found that total tau normalized by GAPDH dramatically decreased (Fig. 8B, C). To learn whether the decrease of tau level is due to reduction of neurons, we measured the level of NeuN, a neuronal marker, by Western blots. We observed a time-dependent reduction of NeuN in KA-injected mouse brains (Fig. 8B). Thus, after normalization with NeuN, the alteration of total tau level was not changed at first 24 hr, but considerably reduced after 36 hr KA-injection (Fig. 8C). Tau phosphorylation at Ser 199 and Ser 396 was dramatically increased and coincided with truncation of GSK-3β (Fig. 8D).

To study the involvement of calpain I activation in truncation of GSK-3β that increases tau phosphorylation, we injected calpeptin, a calpain inhibitor, intracerebroventricularly and then determined the levels of GSK-3β and tau in the KA-treated mice. We found that inhibition of calpain by calpeptin significantly abolished the KA-induced GSK-3β truncation (Fig. 8E). Moreover, the KA-induced increase in tau phosphorylation at Ser 396 was markedly reduced by calpeptin treatment (Fig. 8G, H), even though phosphorylation of GSK-3β at Ser 9 was decreased (Fig. 8E, F). Collectively, these findings suggest that truncation and activation of GSK-3β are caused by activated calpain in excitotoxic brain.

## Discussion

Abnormal hyperphosphorylation of tau is a key lesion in the etiopathogenesis of AD and related tauopathies and is a major therapeutic target. Understanding the underlying molecular mechanisms that lead to this lesion is a major goal in the field of AD and related neurodegenerative disorders. GSK-3β is a major tau protein kinase, but the molecular nature of its involvement in Alzheimer neurofibrillary pathology has not been fully understood. In the present study, we found for the first time that GSK-3β is truncated into a ~41-kDa fragment in AD brain, which increases its tau kinase activity and that over-activation of calpain I is responsible for the GSK-3β truncation. The truncation and activation of GSK-3β is found to be highly and positively correlated with tau phosphorylation and tau pathology in human brain. *In vitro* Calpain I cleaves GSK-3β first into the 43-kDa fragment and further to 41-kDa at the C-terminus and increases the kinase activity toward tau. Proteolysis of GSK-3β by calpain I does not alter its priming characteristic. Like full length GSK-3β, the calpain I-proteolyzed GSK-3β phosphorylates PKA-primed tau more efficiently than control-treated tau. The cleavage site of GSK-3β by calpain I are located at Ser_380_-Ser_381_ of its C-terminus, respectively. Excitotoxicity induced by KA leads to truncation of GSK-3β and increase in tau phosphorylation at GSK-3β favorite sites, Ser 199 and Ser 396 in mouse brain. Collectively, these findings provide direct evidence that truncation of GSK-3β by overactivated calpain I results in the enhancement of its kinase activity and leads to abnormal hyperphosphorylation and pathology of tau in AD brain.

Calpain I is overactivated due to dysregulation of calcium in AD brain. Many putative etiologic factors of AD, including excitotoxicity, β-amyloid neurotoxicity, and free-radical injury, have in common the potential for disrupting intracellular calcium homeostasis^34^,^35^,^36^. Though there is a lack of direct evidence of altered calcium homeostasis in AD brain, dysregulation of calcium is one of the major hypotheses that may explain the pathogenic mechanism of the disease^37^,^38^. Both calpain I and II are present principally as inactive precursors in the cell under basal conditions, and they are activated by calcium-stimulated autoproteolytic cleavage of the N-terminal sequence in response to calcium influx. Calpain I, which is the major calpain isoform in the neuron, is fully activated by low μM concentrations of calcium (hence, it is also called μ-calpain), whereas calpain II requires low mM calcium for optimal activity (hence, also called m-calpain). Beside mM calcium, phosphorylation of calpain II by ERK1/2 can activate it^39^. Calpain is thought to play a critical role in activation of neuronal cdk5^40^,^41^, MAPK pathway^22^, PKA^32^ and protein phosphatase 2B (PP2B)^23^, as well as phosphorylation and truncation of tau^42^, which in turn causes neuronal death^43^,^44^,^45^. Here, we observed that excitotoxicity induced by KA caused truncation of GSK-3β and increased tau phosphorylation at Ser 396 in mouse brains. Inhibition of calpain with calpeptin almost abolished these changes, which further confirms the role of calpain in truncation of GSK-3β and tau phosphorylation at Ser 396. Elevated truncation and activation of calpain I in AD has been previously reported^20^,^23^. The present study demonstrates that calpain I overactivation may also play a role in neurodegeneration via truncation and activation of GSK-3β in AD brain.

In the present study we found that GSK-3β is proteolyzed by calpain I *in vitro*, generating a 41-kDa major truncated form, which reacts with the N-terminal antibodies, R133 (against a.a. 1–13) and anti-pSer9, suggesting that N-terminus of GSK-3β is not chopped out by calpain I and II *in vitro.* The truncation of GSK-3β at N-terminal by calpain II *in vitro* shown previously could be due to overproteolysis^28^,^29^. The truncated GSK-3β in AD was detected by anti-N-terminal antibodies, supporting the truncation at the C-terminus. Both truncated GSK-3β in AD brain and calpain I-truncated GSK-3β *in vitro* immunoreact with R127, but not G7914, indicating they share similar truncation sites, which are located within the region between these two antibodies' epitopes, amino acid 377–416. Moreover, here, we found that the C-terminus of GSK-3β is truncated out in AD brain and calpain I may be responsible for this truncation. However, we can not exclude the involvement of calpain II in this event. In the present study, we found that proteolysis of GSK-3β by calpains I and II was similar. Removal of C-terminus (a.a. 382–433) of GSK-3β by calpain I markedly enhances its kinase activity towards tau, confirming an autoinhibitory domain at the C-terminus^29^. Patients with Parkinson's disease revealed a disease-related SNP site that is associated with altered splicing of exons 9 and 11. Exon 9 and 11 encode residues 304–316 and 379–411, respectively. Exclusion of exon 9 and/or 11 enhances GSK-3β kinase activity to tau^46^,^47^. Taken together, the C-terminus of GSK-3β appears to be an inhibitory domain and removal of the C-terminus by calpain I enhances its kinase activity.

GSK-3β is a major tau kinase involved in the phosphorylation of tau. GSK3β phosphorylates tau at various sites that are hyperphosphorylated in AD brain^7^,^27^. Inhibition of GSK-3β reduces tau phosphorylation^48^,^49^,^50^. Overexpression of GSK-3β in animals promotes the hyperphosphorylation of tau and accelerates tau-induced neurodegeneration^11^,^12^,^13^,^17^, while the inhibition of its activity reduces tau toxicity and rescues neurodegeneration^14^,^15^,^16^,^17^. GSK-3β phosphorylates tau at many sites with variable efficiencies^27^ and pre-phosphorylation of tau by PKA^27^ and Dyrk1A^51^ promotes its further phosphorylation at most of the tau sites by GSK-3β. In this study, we found that proteolyzed GSK-3β had a higher kinase activity to phosphorylate tau site-specifically and PKA-primed tau was also a better substrate for the proteolyzed GSK-3β, indicating that the truncation of GSK-3β does not affect the priming characteristic of the kinase. We found that the priming of tau by PKA inhibited its phosphorylation at Thr 212 by both full length and the truncated GSK-3β. This negative priming effect is most probably due to the proximity of Thr 212 to the PKA major site, Ser 214. Hence, the truncation of GSK-3β is highly and positively correlated with tau phosphorylation at many sites in human brains, indicating increased tau phosphorylation in AD brain may result from increased GSK-3β kinase activity caused by its C-terminal truncation by calpain I. Activation of calpains might also involve truncation and activation or inhibition of other tau protein kinases or phosphatses and directly or indirectly affect tau phosphorylation. In the present study, we cannot rule in or rule out such other pathways to tau pathology.

GSK-3β also regulates tau pre-mRNA splicing^52^ and expression^53^, which may be associated with tau pathogenesis. In addition to tau metabolism, GSK-3β interferes with the biology of Aβ, another critical molecule in the pathogenesis of AD. The amyloid precursor protein (APP) and presenilin are substrates of GSK-3β^25^,^54^,^55^. Aβ was reported to promote tau phosphorylation and accelerate tau pathology by several mechanisms, including activation of GSK-3β^56^,^57^,^58^,^59^. Genetic or pharmacological inactivation of GSK-3β reduces Aβ and its associated toxicity, ameliorates Aβ-induced behavioral deficits, and rescues neuronal loss in AD mouse models^60^,^61^,^62^. Thus, these data strongly implicate overactivation of GSK-3β in the pathogenesis of AD.

It is reported that activation of extrasynaptic NMDA receptor, but not synaptic NMDA receptor, causes activation of calpain^63^. Soluble Aβ oligomers inhibit long-term potentiation through activation of extrasynaptic NR2B-containing NMDA receptors. The calpain inhibitor calpeptin significantly rescues the soluble Aβ effect on LTP^64^. Therefore, Aβ may act on extrasynaptic NMDA receptor and activate calpain, leading to truncation and activation of GSK-3β, consequently, contributing to hyperphosphorylation of tau and tau pathology. Memantine, a NMDA receptor antagonist, is an approved drug for the treatment of moderate-to-severe AD^65^, which suggests the involvement of dysregulation of calcium/calpain in AD. However, memantine only has a modest effect in moderate-to-severe Alzheimer's disease^66^, and does not appear to be effective in mild disease^67^, indicating that overactivation of calpain I and GSK-3β truncation might appear in advanced stage of the disease.

In summary, the present study provides a direct evidence that GSK-3β is truncated by overactivated calpain I at the C-terminus in AD brain, resulting in increased kinase activity. The truncated GSK-3β phosphorylates tau much more efficiently and causes tau hyperphosphorylation and neurofibrillary degeneration in AD. These studies provide a novel molecular mechanism by which calpain I overactivation through truncation and activation of GSK-3β leads to abnormal hyperphosphorylation of tau and neurodegeneration in AD.

## Methods

### Human brain tissues

Medial frontal cortical tissue samples from seven AD and seven age-matched normal human brains used for this study (Table S1) were obtained from the Sun Health Research Institute Donation Program (Sun City, AZ). All brain samples were from histopathologically confirmed cases and were stored at −70°C until used. The use of frozen human brain tissue was in accordance with the National Institutes of Health guidelines and was approved by our institute's institutional review committee.

### Animals

Male FVB mice were purchased from Charles River Laboratory or Model Animal Research Center of Nanjing University. The animals were housed in a 12-hour light/dark schedule with free access to food and water. Animal use was in full compliance with the NIH guidelines and was approved by our institutional Animal Care and Use Committees.

### Plasmids, proteins, and antibodies

pRK172 containing the largest isoform of human tau, pRK172/tau_441_, was kindly provided by Dr. Michel Goedert (Molecular Biology Unit, Medical Research Council, Cambridge, U.K.). pcDNA/GSK-3β was a kind gift from Dr. Jesús Avila (Centro de Biología Molecular, Universidad Autónoma de Madrid, Madrid, Spain), from which pCI/HA-GSK-3β was constructed and its sequence was confirmed. Recombinant tau_441_ was expressed and purified from *E.* coli in our laboratory. Recombinant GSK-3β and calpain II and N-acetyl-Leu-Leu-Nle-CHO (ALLN) were bought from Calbiochem (San Diego, CA). Calpain I and the catalytic subunit of protein kinase A were bought from Sigma (St. Louis, MO). Primary antibodies used in this study are listed in Table S2. Horseradish peroxidase (HRP) conjugated anti-mouse and anti-rabbit IgG were obtained from Jackson ImmunoResearch Laboratories (West Grove, PA). ECL (enhanced chemiluminescence) Kit was from Thermo Scientific (Rockford, IL), and [Υ-^32^P] ATP was from MP Biomedicals (Irvine, CA).

### Western blots analyses

Human Brain tissue was homogenized in 9 volumes of buffer containing 50 mM Tris-HCl, pH 7.4, 8.5% sucrose, 10 mM β-mercaptoethanol, and 2.0 mM EDTA. After adding 2-fold concentrated Laemmli buffer, the brain homogenates were boiled for 5 min and the protein concentration was measured by using modified Lowry. The same amounts of protein from each sample were separated by sodium dodecyl sulfate (SDS)–polyacrylamide gel electrophoresis (PAGE) and electro-blotted onto PVDF membrane. After blocking with 5% fat-free milk, the membrane was incubated with primary antibodies, such as anti-GSK-3β (1:1000), anti-calpain I (1:5000), anti-GAPDH (1:2000), or anti-pSer9-GSK-3β (1:1000), overnight at room temperature in the presence of 0.01% NaN_3_. After washing with TBST (Tris-HCl, pH 7.4, 150 mM NaCl, 0.05% Tween 20) three times, the membrane was incubated with the corresponding HRP-conjugated secondary antibody for ~2 h. After washing with TBST, the blot was visualized by enhanced chemiluminescence (Thermo Scientific, Rockford, IL) and quantified by densitometry and AIDA 2.0 beta software (Raytest, Straubenhardt, Germany).

### Immuno-dot blots analyses

To measure phosphorylated tau level in the brain crude extracts or in the phosphorylation products, the samples were diluted serially with 0.2% BSA in TBS containing 50 mM NaF, 1 mM Na_3_VO_4_, and 2 μg/ml each of aprotinin, leupeptin, and peptstatin and applied onto nitrocellulose membrane (Schleicher and Schuell, Keene, NH) at 5 μl/grid (7 × 7 mm). The blot was placed in a 37°C oven for 1 h to allow the protein to bind to the membrane, and processed as described above for Western blots.

### Affinity-purification of HA tagged GSK-3β at N-terminus (HA-GSK-3β) or GST fusion GSK-3β at C-terminus (GSK-3β-GST) from HEK-293FT cells

HEK-293FT cells were maintained in Dulbecco's modified Eagle's medium (DMEM) supplemented with 10% fetal bovine serum (Invitrogen, Carlsbad, CA) at 37°C and transfected with pCI/HA-GSK-3β or pCI/GSK-3β-GST with FuGENE 6 (Roche Applied Science, Indianapolis, IN) according to the manufacturer's manual. After 48 h transfection, the cells were washed twice with PBS, and lysed with lysis buffer (50 mM Tris-HCl, pH 7.4, 150 mM NaCl, 50 mM NaF, 1 mM Na_3_VO_4_, 0.1% NP-40, 0.1% Triton X-100, 0.2% Sodium deoxycholate, 2 mM EDTA, 10 mM β-mercaptoethanol, 5 mM AEBSF, 10 μg/ml aprotinin, 10 μg/ml leupeptin and 10 μg/ml pepstatin). Insoluble materials were removed by brief centrifugation at 16,000 × g. The supernatant was incubated with anti-HA antibody pre-conjugated onto protein G beads overnight or with glutathione conjugated beads for 1 h at 4°C. The beads were washed with lysis buffer twice and with TBS twice, and affinity purified HA-GSK-3β or GSK-3β-GST was confirmed by Western blots and subjected to proteolysis or kinase activity assay.

### Preparation of PKA-phosphorylated tau (PKA-tau)

PKA-phosphorylated tau was prepared as described previously^27^. Briefly, recombinant tau_441_ (1.0 mg/ml) was incubated with the catalytic subunit of PKA (10 mg/ml) in the buffer consisting of 40 mM HEPES (pH 6.8), 10 mM β-mercaptoethanol, 10 mM MgCl_2_, 1.0 mM EGTA and 0.2 mM ATP for 60 min. The reaction was terminated by adding trichloroacetic acid to the reaction mixture to a final concentration of 8%. The precipitated tau was collected by centrifugation and washed with ethanol. The dried tau pellets were then reconstituted in 5.0 mM 2-(N-morpholino) ethanesulfonic acid monohydrate (MES), pH 6.8, containing 0.1 mM EGTA. The control tau was treated the same way in parallel except that kinase was added to the mixture after the addition of trichloroacetic acid.

### In vitro proteolysis of GSK-3β

Recombinant GSK-3β (Calbiochem, San Diego, CA) or affinity purified GSK-3β, as described above, was proteolyzed *in vitro* by incubating with calpain I (Sigma) or calpain II (Calbiochem) in proteolysis buffer (50 mM Tris-HCl, pH 7.4, 1 mM CaCl_2_, 10 mM β-mercaptoethanol) for 10 min at 30°C. After termination of proteolysis by adding 50 μM ALLN (a cysteine protease inhibitor) to stop the proteolysis, the proteolyzed products were subjected for kinase activity assay or Western blot analyses.

Proteolysis of GSK-3β in human brain homogenates is performed as described previously^23^. Briefly, human brain tissue was homogenized in 9 volumes of buffer consisting of 50 mM Tris-HCl, pH7.4, 8.5% sucrose, 10 mM β-mercaptoethanol and 2.0 mM EDTA, followed by centrifugation at 16,000 × g at 4°C for 10 min. The supernatants were incubated in the presence or absence of various concentrations of Ca^2+^ and/or protease inhibitors for 10 min at 30°C. The reactions were terminated by addition of 4-fold concentrated SDS-PAGE sample buffer, followed by heating in boiling water for 5 min. The products of proteolysis were analyzed by Western blots developed with antibodies to calpain I and GSK-3β.

### GSK-3β kinase activity assay

For measuring the activity of GSK-3β, tau_441_ (0.2 mg/ml) was incubated with GSK-3β samples in a reaction buffer consisting of 50 mM Tris-HCl (pH 7.4), 10 mM β-mercaptoethanol, 0.1 mM EGTA, 10 mM MgCl_2_ and 0.2 mM [γ-^32^P]ATP (500 cpm/pmol). After incubation at 30°C for 30 min, the reaction was stopped by applying the reaction mixture on a chromatography paper strip pre-spotted with 10% trichloroacetic acid, the ^32^P-labeled tau was separated from free [γ-^32^P]ATP by paper chromatography, and the radioactivity of tau was determined by Cerenkov counting, as described previously^27^.

### Site-specific phosphorylation of tau by cleaved GSK-3β

Immunopurified GSK-3β with anti-HA as described above was proteolyzed *in vitro* by incubating with 2 μg/ml calpain I (Sigma) in proteolysis buffer (50 mM Tris-HCl, pH 7.4, 1 mM CaCl_2_, 10 mM β-mercaptoethanol) for 10 min at 30°C. After termination of proteolysis by adding 50 μM ALLN, the proteolyzed GSK-3β was incubated with tau_441_ (0.2 mg/ml) in a reaction buffer consisting of 50 mM Tris-HCl (pH 7.4), 10 mM β-mercaptoethanol, 0.1 mM EGTA, 10 mM MgCl_2_ and 0.2 mM ATP. After incubation at 30°C for various times, the reaction was stopped by adding acetic acid. The reaction products were subjected to immuno-dot blot analysis for the site-specific phosphorylation of tau, as described previously^7^.

### Kainic acid injection of mice

Male FVB mice (25–30 g body weight, 12 weeks old) were housed individually in a 12-hour light/dark schedule with free access to food and water. A single dose of kainic acid (KA, 20 mg/kg body weight) was injected intraperitoneally^32^. The mice were then sacrificed at 2.5, 6, 10, 24 and 36 hr after injection, and forebrains were immediately removed and stored at −80°C.

For calpain inhibition, above FVB mice were injected intracerebroventricularly with calpeptin. For detail, the mice were first anesthetized by intraperitoneal injection of Avertin (Sigma) and placed on a stereotactic instrument. After the scalp was incised and retracted, a 10 μl syringe (Hamilton) was stereotactically placed into the lateral ventricle of the cerebrum according to stereotaxic coordinates (bregma and dura of anterior–posterior 0.5 mm, left lateral 1.0 mm and dorsal–ventral 2.5 mm). A total of 2 μl of 2 μM calpeptin dissolved in DMSO was injected into the left ventricle of the brain. The same volume of DMSO was injected into the left ventricle for control animals. Mice were treated with KA 3 hr after the injection.

### Statistical analysis

Data were presented as mean ± S.D. and analyzed by the unpaired two-tailed Student's *t* test or Mann Whitney test (for the data with non-normal distribution) for two groups comparison and by one way ANOVA for multiple-groups analysis. The Spearman correlation coefficient r was calculated to compare the correlation between calpain I activation and the GSK-3β truncation or between GSK-3β truncation and tau phosphorylation.

## Author Contributions

N.J., X.Y. and D.Y. performed experiments and analyzed data; M.C., F.D. and X.G. provided reagents and participated in discussions; C.X.G. and K.I. provided reagents and reviewed the paper critically; and F.L. performed proteolysis *in vitro*, kinase activity assay and immune-blot analyses, designed the study, analyzed data and wrote the paper.

## Supplementary Material

Supplementary Information Truncation and activation of GSK-3β by calpain I: a molecular mechanism links to tau hyperphosphorylation in Alzheimer's disease
