# Supplementary material for: Truncation and activation of GSK-3β by calpain I: a molecular mechanism links to tau hyperphosphorylation in Alzheimer's disease
Source: Sci Rep. 2015 Feb 2;5:8187. doi: 10.1038/srep08187 (PMC4313118; doi:10.1038/srep08187)
Supplement: Supplementary Information — Truncation and activation of GSK-3β by calpain I: a molecular mechanism links to tau hyperphosphorylation in Alzheimer's disease [file srep08187-s1.doc]

Truncation and activation of GSK-3β by calpain I: a molecular mechanism links to tau hyperphosphorylation in Alzheimer’s disease

**Nana Jin^1,2,*^, Xiaomin Yin^1,2,*^, Dian Yu^2^, Maohong Cao^3^, Cheng-Xin Gong^2^, Khalid Iqbal^2^, Fei Ding^1^, Xiasong Gu^1^, Fei Liu^1,2^**

*^1^Jiangsu Key Laboratory of Neuroregeneration, Co-innovation Center of Neuroregeneration, Nantong University, Nantong, Jiangsu 226001, P. R. China.*

^2^Department of Neurochemistry, New York State Institute for Basic Research in Developmental Disabilities, Staten Island, New York 10314, USA.

*^3^Institute of Neurology, Department of Neurology, Hospital Affiliated to Nantong University, Nantong, Jiangsu 226001, P. R. China.*

*Correspondence and requests for materials should be addressed to F. L. (feiliu63@hotmail.com).*

**Equally contributed to this work*

Running title: Truncation and activation of GSK-3β by calpain I

**SUPPLEMENTAL INFORMATION**

**EXPERIMENTAL PROCEDURES**

**Effect of postmortem delay on the truncation of GSK-3β.** Adult C57B6 mice (5 months old) were killed by CO_2_ chamber. The dead bodies were kept at room temperature or 4°C for various times. The forebrains were analyzed by Western blots.

**Table S1. Alzheimer’s disease (AD) and control (Con) cases used in this study**

^a^ PMI = postmortem interval; ^b^ Neurofibrillary pathology was staged according to Braak and Braak ; ^c^ Tangle score was a density estimate and was designated as none, sparse, moderate or frequent (0, 1, 2 or 3 for statistics), as defined according to CERAD Alzheimer disease criteria . Five areas (frontal, temporal, parietal, hippocampal and entorhinal) were examined, and the scores were combined for a maximum of 15.

| Case | Age at death (year) | Gender | PMI^a^ (h) | Braak stage^b^ | Tangle scores^c^ |
| --- | --- | --- | --- | --- | --- |
| AD 1 | 89 | F | 3 | V | 14.5 |
| AD 2 | 80 | F | 2.25 | VI | 14.5 |
| AD 3  AD 4 | 85  78 | F  F | 1.66  1.83 | V  VI | 12.0  15.0 |
| AD 5 | 95 | F | 3.16 | VI | 10.0 |
| AD 6 | 86 | M | 2.25 | VI | 13.5 |
| AD 7 | 91 | F | 3 | V | 8.50 |
| Mean ± SD | 86.29 ± 5.99 |  | 2.45 ± 0.61 |  | 12.57 ± 2.51 |
| Con 1 | 85 | M | 25 | II | 4.25 |
| Con 2 | 86 | F | 2.5 | III | 5.00 |
| Con 3 | 81 | M | 2.75 | III | 6.41 |
| Con 4 | 88 | F | 3 | II | 2.00 |
| Con 5 | 90 | F | 3 | III | 4.50 |
| Con 6 | 88 | F | 3.5 | III | 2.50 |
| Con 7 | 88 | F | 3 | IV | 4.50 |
| Mean ± SD | 86.6 ± 2.9 |  | 2.89 ± 0.39 |  | 4.17 ± 1.50 |

**Table S2. Primary antibodies employed in this study**

| **Antibody** | **Type** | **Specificity** | **Phosphorylation site/ Epitope** | **Reference/Source** |
| --- | --- | --- | --- | --- |
| R134d | Poly- | Tau | N/A |  |
| 43D | Mono- | tau | 15-32 |  |
| Anti-pS199-tau | Poly- | p-tau | pSer199 | Invitrogen, Carlsbad, CA |
| Anti-pS202-tau | Poly- | p-tau | pSer202 | Invitrogen, Carlsbad, CA |
| Anti-pT205-tau | Poly- | p-tau | pThr205 | Invitrogen, Carlsbad, CA |
| Anti-pT212-tau | Poly- | p-tau | pThr212 | Invitrogen, Carlsbad, CA |
| Anti-pS214-tau | Poly- | p-tau | pSer214 | Invitrogen, Carlsbad, CA |
| Anti-pT217-tau | Poly- | p-tau | pThr217 | Invitrogen, Carlsbad, CA |
| 12E8 | Mono- | p-tau | pSer262 | Invitrogen, Carlsbad, CA |
| Anti-pS396-tau | Poly- | p-tau | pSer396 | Invitrogen, Carlsbad, CA |
| Anti-pS404-tau | Poly- | p-tau | pSer404 | Invitrogen, Carlsbad, CA |
| R145d | Poly- | p-tau | pSer422 |  |
| Anti-pSer9-GSK-3β | Poly- | p-GSK-3β | pSer9 | Cell Signaling, MA |
| Anti-pTyr216-GSK-3β | Poly- | p-GSK-3β | pTyr216 | Invitrogen, Carlsbad, CA |
| R133d | Poly- | GSK-3α/β | a.a. 1-13 |  |
| R127d | Poly- | GSK-3β  | a.a. 364-377 | Produced in our laboratory |
| Anti-GSK-3β (11B9)  | Mono- | GSK-3β  | N-terminus | Santa-Cruz, Santa Cruz, CA |
| Anti-GSK-3α/β (D75D3) | Poly- | GSK-3α/β | ~Gln269 | Cell Signaling, MA |
| Anti-GSK-3β (27C10) | Poly- | GSK-3β  | C-terminus | Cell Signaling, MA |
| Ant-GSK-3β (1F7) | Poly- | GSK-3β  | a.a. 355-433 | Santa-Cruz, Santa Cruz, CA |
| Anti-GSK-3β (G7914) | Poly- | GSK-3β  | a.a. 416-433 | Sigma, St. Louis, MO |
| Anti-calpain I | Poly- | Calpain I | Domain II | Calbiochem, La Jolla, CA |
| Ant-HA | Poly- | HA |  | Sigma, St. Louis, MO |
| Anti-HA | Mono- | HA |  | Sigma, St. Louis, MO |
| Anti-actin | Mono- | Actin |  | Sigma, St. Louis, MO |
| Anti-GAPDH | poly- | GAPDH |  | Santa-Cruz, Santa Cruz, CA |

Abbreviations: GAPDH, glyceraldehyde-3-phosphate dehydrogenase; GSK-3β, glycogen synthase kinase-3β; mono-, monoclonal; p-, phosphorylated; Poly-, polyclonal; Ser, serine; Thr, threonine; Tyr, tyrosine.


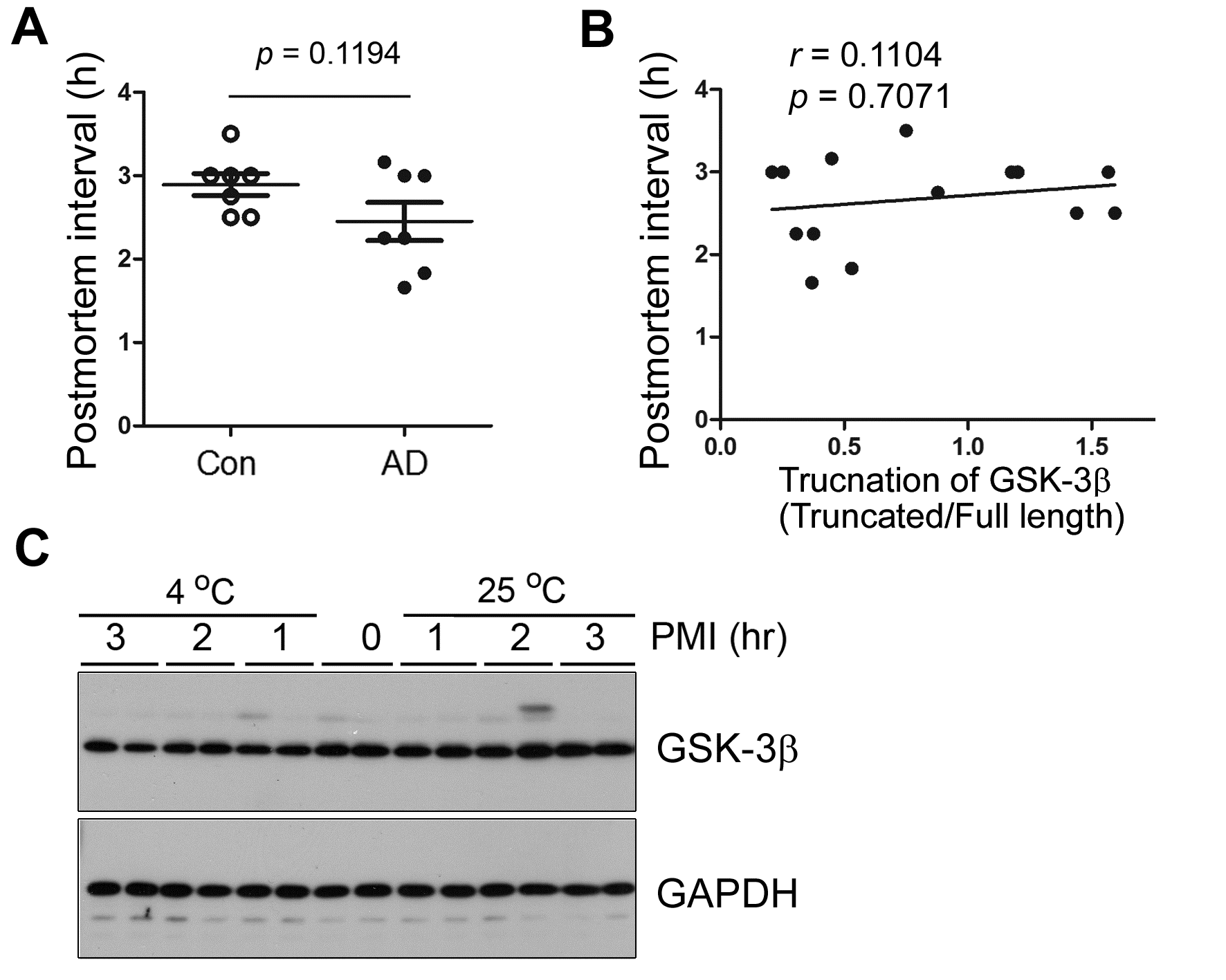


**Figure S1. Postmortem interval (PMI) up to 3 hours studied does not affect the truncation of GSK-3β.** (A) The PMI of the AD cases used in this study is a little shorter than control cases but there is no statistically significant difference between the two groups. Data are presented as mean ± S.D. (B). There is no significant correlation between GSK-3β truncation and postmortem delay (hr) in AD and control cases studied. The truncation (truncated/full length) of GSK-3β in human brains (from Figure 1) was plotted against postmortem interval and analyzed with Pearson correlation analysis. (C) Western blots developed with anti-N-terminal GSK-3β, 11B9, shows no detectable truncation of GSK-3β in the brain upto three hours postmortem interval in mice whose bodies were stored at 4 ^o^C or 25 ^o^C for 0, 1, 2 and 3 hours postmortem.


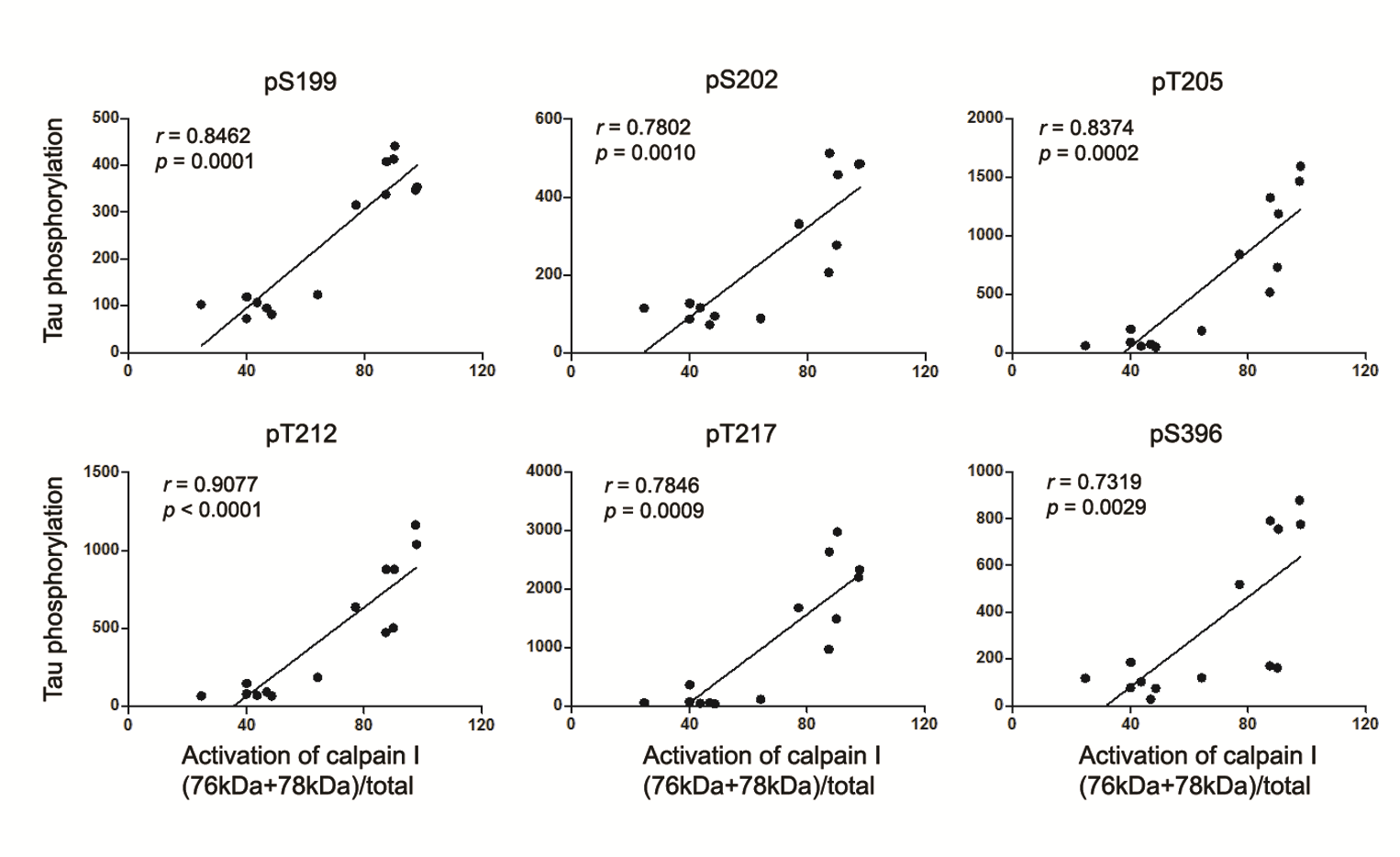


**Figure S2.** Activation of calpain I is positively correlated with tau phosphorylation in human brain. Level of activation of calpain I in frontal cortical homogenates from AD and control cases determined by Western blots (Figure 1) and tau phosphorylation at individual phosphorylation sites in the frontal cortical crude extracts from quantitative immuno-dot-blots (not shown) were quantified by densitometry. The levels of tau phosphorylation at individual phosphorylation sites (Y-axis) were then plotted against the truncation/activation of calpain I (ratio of the truncated over the total calpain I) (X-axis).


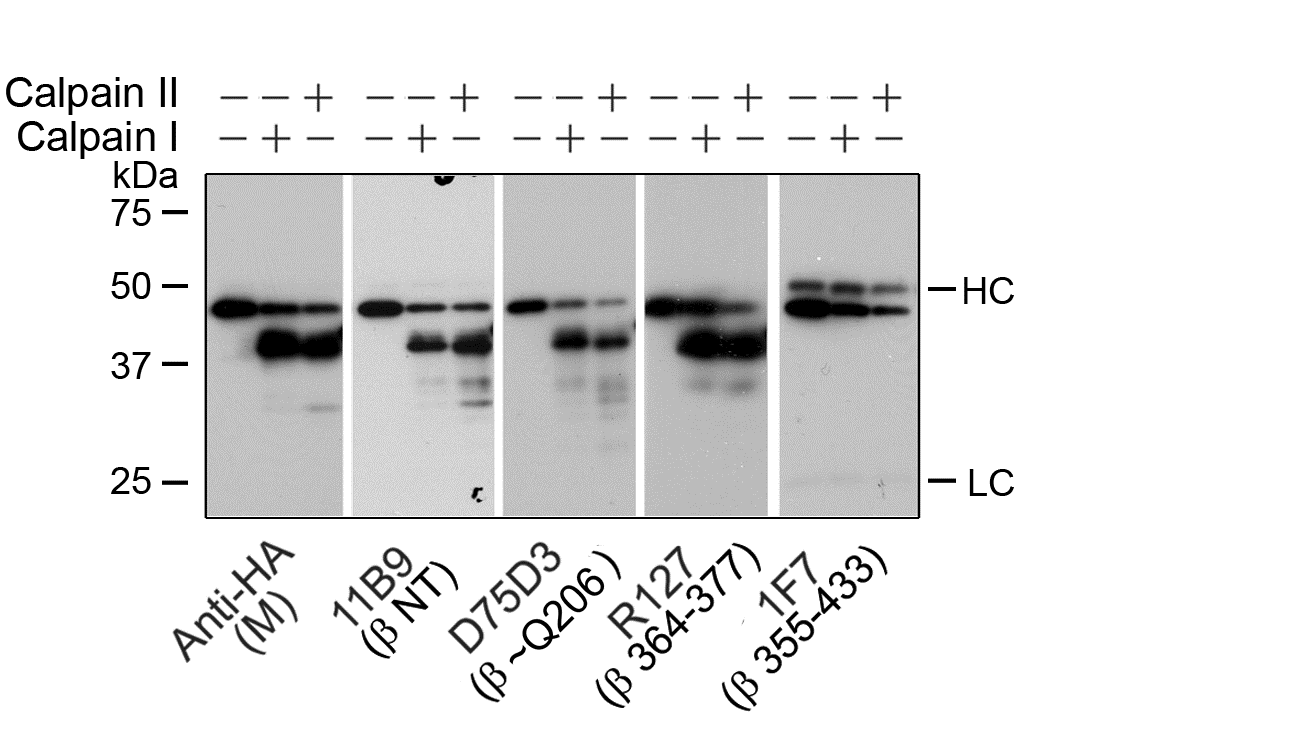


**Figure S3. GSK-3β is truncated *in vitro* by calpains I and II similarly.** Western blots of immuno-purified **r**ecombinant HA tagged GSK-3β from HEK-293FT cells by anti-HA in vitro proteolyzed with calpain I or II. HA-GSK-3β was immunoprecipitated using anti-HA and incubated with or without 0.2 μg/ml calpain I or calpain II in the presence of 1 mM CaCl_2_ for 10 min at 30^o^C. The reaction products were detected by Western blots developed with anti-HA or different anti-GSK-3β as labeled under the blots.


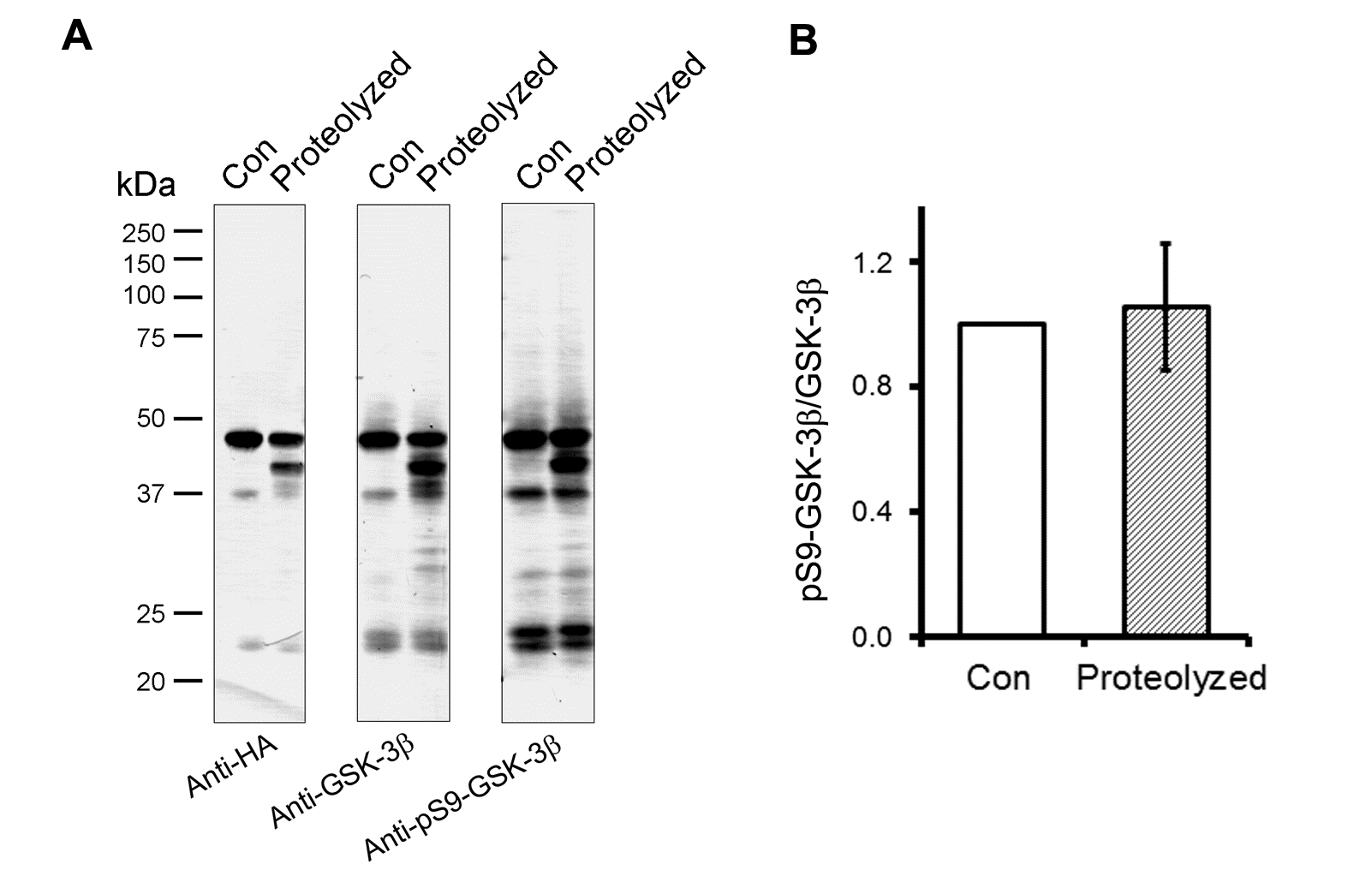


**Figure S4. Proteolysis of GSK-3β by calpain I *in vitro* does affect its phosphorylation at Ser 9.** (A) Western blots and (B) densitometric quantification of the blots of proteolysis of immune-purified GSK-3β by calpain I. Immuno-purified GSK-3β by anti-HA from HEK-293FT cells by anti-HA was incubated with or without 0.2 μg/ml calpain I in the presence of I mM CaCl_2_ for 10 min at 30^o^C. The level of phosphorylated GSK-3β is normalized with total GSK-3β and presented as mean ± S.D. (n=3).


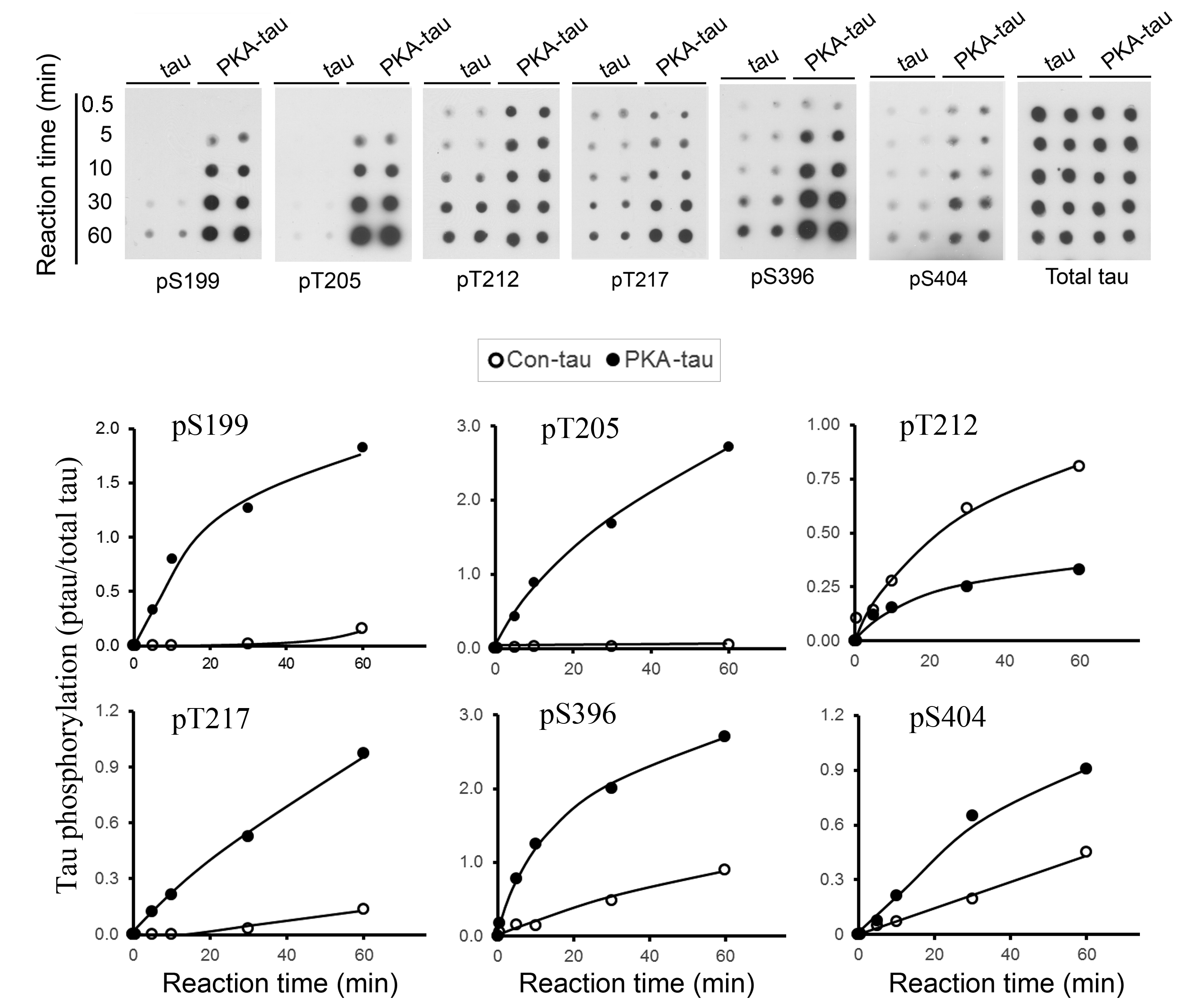


**Figure S5. GSK-3β phosphorylates PKA-phosphorylated tau more efficiently than the non-phosphorylated protein at several sites except at Thr 212.** PKA-pre-phosphorylated recombinant tau_441_ was prepared as described in materials and methods. Immunopurified GSK-3β from mammalian cells was incubated with tau_441_ or PKA-phosphorylated tau_441_ for various times. The phosphorylation level of tau at individual sites was detected by immuno-dot blots developed with different phospho-dependent and site-specific and total tau antibodies (**A**) and plotted against the phosphorylation reaction time (**B**) after densitometric quantification and normalization with total tau.


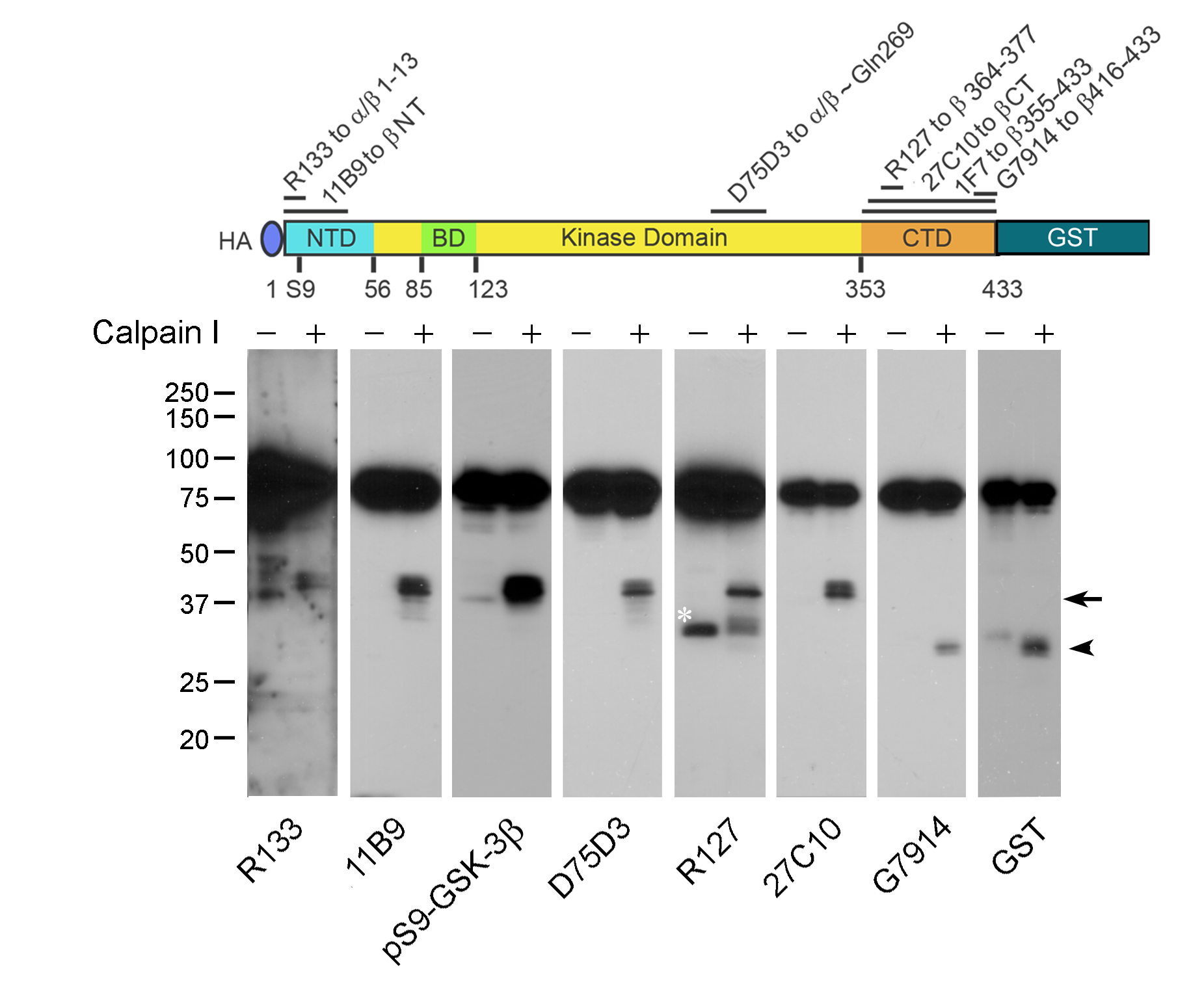


**Figure S6. GSK-3β-GST is truncated by calpain I *in vitro*.** (A) Schematic of GST-fused GSK-3β showing different domains and sites of different antibodies to GSK-3β. (B) Western blots of GSK-3β-GST fusion protein proteolyzed with calpain I. Recombinant GSK-3β-GST was incubated without or with 0.2 μg/ml calpain I in the presence of 1 mM CaCl_2_ for 10 min at 30^o^C. The proteolysed products were subjected to Western blots developed with antibodies against different epitopes of GSK-3β indicated in (A). Arrow indicates N-terminal portion of GSK-3β and arrow head indicates C-terminal portion of GSK-3β fused with GST. * indicates non-proteolysis-related-band.

**References:**

1. Braak H, Braak E. Staging of Alzheimer's disease-related neurofibrillary changes. Neurobiol Aging 1995; 16: 271-278; discussion 278-284.

2. Liu F, Shi J, Tanimukai H, Gu J, Gu J, Grundke-Iqbal I, Iqbal K, Gong CX. Reduced O-GlcNAcylation links lower brain glucose metabolism and tau pathology in Alzheimer's disease. Brain 2009; 132: 1820-1832.

3. Mirra SS, Heyman A, Mckeel D, Sumi SM, Crain BJ, Brownlee LM, Vogel FS, Hughes JP, Van Belle G, Berg L. The Consortium to Establish a Registry for Alzheimer's Disease (CERAD). Part II. Standardization of the neuropathologic assessment of Alzheimer's disease. Neurology 1991; 41: 479-486.

4. Pei JJ, Tanaka T, Tung YC, Braak E, Iqbal K, Grundke-Iqbal I. Distribution, levels, and activity of glycogen synthase kinase-3 in the Alzheimer disease brain. J Neuropathol Exp Neurol 1997; 56: 70-78.

5. Pei JJ, Gong CX, Iqbal K, Grundke-Iqbal I, Wu QL, Winblad B, Cowburn RF. Subcellular distribution of protein phosphatases and abnormally phosphorylated tau in the temporal cortex from Alzheimer's disease and control brains. J Neural Transm 1998; 105: 69-83.
